# Striatonigrostriatal Spirals in Addiction

**DOI:** 10.3389/fncir.2021.803501

**Published:** 2021-12-10

**Authors:** Andy Sivils, John Q. Wang, Xiang-Ping Chu

**Affiliations:** Department of Biomedical Sciences, School of Medicine, University of Missouri-Kansas City, Kansas City, MO, United States

**Keywords:** striatonigrostriatal spirals, addiction, reward system, basal ganglia circuits, neurotransmitter, dopamine, behavior

## Abstract

A biological reward system is integral to all animal life and humans are no exception. For millennia individuals have investigated this system and its influences on human behavior. In the modern day, with the US facing an ongoing epidemic of substance use without an effective treatment, these investigations are of paramount importance. It is well known that basal ganglia contribute to rewards and are involved in learning, approach behavior, economic choices, and positive emotions. This review aims to elucidate the physiological role of striatonigrostriatal (SNS) spirals, as part of basal ganglia circuits, in this reward system and their pathophysiological role in perpetuating addiction. Additionally, the main functions of neurotransmitters such as dopamine and glutamate and their receptors in SNS circuits will be summarized. With this information, the claim that SNS spirals are crucial intermediaries in the shift from goal-directed behavior to habitual behavior will be supported, making this circuit a viable target for potential therapeutic intervention in those with substance use disorders.

## Introduction

Addiction is defined as a “chronic, relapsing disorder characterized by compulsive drug seeking, continued use despite harmful consequences, and long-lasting changes in the brain” (Goldstein and Volkow, 2011; Koob, 2021; National Institute on Drug Abuse, 2021). Initially, these behaviors of consumption are motivated by the experience of a reward. Clinically, disorder is noted when that consumption continues in the face of negative results with concomitant reductions in the individual’s quality of life (American Psychiatric Association, 2013). In other words, when the reward stops outweighing the objective costs of the action, addiction begins.

According to the Centers for Disease Control and Prevention (CDC), the United States is experiencing a drug overdose epidemic (Centers for Disease Control and Prevention, 2021). A total of 128 people die every day from a drug overdose, and the number of drug overdoses in 2018 was four times the number in 1999 (Centers for Disease Control and Prevention, 2021). Strikingly, drug overdose is now the leading cause of injury-related death (Brooks, 2020). These data underscore the importance for continued research investigating the mechanisms that lead to this deadly disease. In addition to the concern based on the rising number of fatalities, there is not standard therapeutic treatment for addiction that is effective. Project MATCH was a multi-scale clinical trial designed to test a series of hypotheses on how patient-treatment interactions relate to outcomes (Mattson et al., 1993). In the data from Project MATCH, correlations between treatment attendance and outcome were small—accounting for only 3% of the variance (Cutler and Fishbain, 2005). In order to better delineate the mechanisms that underpin the development of addiction, new treatment opportunities are critical for therapeutic discovery, which would address this important issue.

Contemporary neuroscience has made great progress in describing the reward system affected in those with addiction (Lüscher et al., 2020). It is generally accepted that Pavlovian and instrumental learning mechanisms, which are supported by basal ganglia and limbic system neural connections, overpower natural prefrontal inhibition and lead to the reduction in behavioral control observed in patients with addiction (Belin et al., 2009; Koob, 2021). It is thought that the transition from healthy consumption to pathology is facilitated by neural circuitry changes within the basal ganglia network (Alexander et al., 1986), which is organized into parallel cortico-striato-pallido-cortical loops (Alexander and Crutcher, 1990; Everitt and Robbins, 2005; Everitt and Robbins, 2016; Florio et al., 2018). Within this circuitry, the ventral striatum (VS) is pinned as the major interface between emotion, motivation, and action—a major component of the ventral medial striatum (VMS) being the nucleus accumbens (NAc) (Mogenson et al., 1980; Scofield et al., 2016). In fact, all drugs of abuse commonly impact the NAc by mostly increasing its dopaminergic and/or glutamatergic transmission (Chiara and Imperato, 1988; Liu and Kaeser, 2019; Buck et al., 2021). This region is split into a core and shell, and the core is specifically noted to potentiate Pavlovian-instrumental transfer which is the basic mechanism of stimuli associated with reward altering motivational salience and operant behavior (Everitt and Robbins, 2005; Meredith et al., 2008).

If a person experienced a reward and wanted to change their behavior to increase the amount of reward they experienced, there wouldn’t be a problem. Where the issue would, and does, arise is whenever the drive to achieve that reward becomes habitual past the point of rational decision making. Here, there is nuance within the dorsal striatum (DS) (Belin et al., 2009; Redgrave et al., 2010). It is noted that the dorsomedial striatum (DMS) regulates goal-directed processing while the dorsolateral striatum (DLS) regulates habitual control (Belin et al., 2009; Redgrave et al., 2010). These individual subsystems of the striatum facilitate different levels of learning and behavior, with goal-directed behavior eventually turning into habitual control with enough stimulus over time (Belin et al., 2009; Redgrave et al., 2010).

Importantly, dopaminergic transmission within the striato-nigro-striatal (SNS) ascending spirals come from the brainstem to the NAc and reach to the more dorsal regions of the striatum, influencing these shifts from action-outcome to stimulus-response processing (Haber et al., 2000; Belin et al., 2009; Bamford et al., 2018). For example, the successful operation of Pavlovian instrumental transfer has been reported to depend upon the DLS, but not the DMS (Corbit and Janak, 2007; Belin et al., 2009). Without these SNS spirals, evidence suggests that individuals would not be able to transition from goal directed behavior to habitual control like is seen in the common example of driving the same route to work each day (Yin and Knowlton, 2006). Thus, further summary of the anatomy and function of these spirals is worthy of review.

## SNS Circuit

The anatomy of the SNS spirals is complicated due to the nature of the brain region they are within, and their connections to different tissues within said region (Haber et al., 2000). That region is the basal ganglia, which had its functions first examined at the beginning of 20th century due to clinical observations of patients with lesions (Redgrave et al., 2010; Lanciego et al., 2012). These investigations eventually led to the classification of the basal ganglia, which includes the DS, VS, globus pallidus, substantia nigra (SN), ventral tegmental area (VTA), and subthalamic nuclei if reciprocally connected regions are accounted for (Packard and Knowlton, 2002). Originally, all these basal ganglia components were considered to function mainly as a way station for motor movement. Information would pass from the cortex, through the basal ganglia, and then back to the cortex to either produce or inhibit actions (Packard and Knowlton, 2002). As the century progressed, the basal ganglia began to be seen as something more than a motor function area. With new neuronal tracing techniques perfected by Nauta and Gygax (1954) and Packard and Knowlton (2002) in the mid-1950s, further specifications within the basal ganglia became apparent.

**FIGURE 1:**
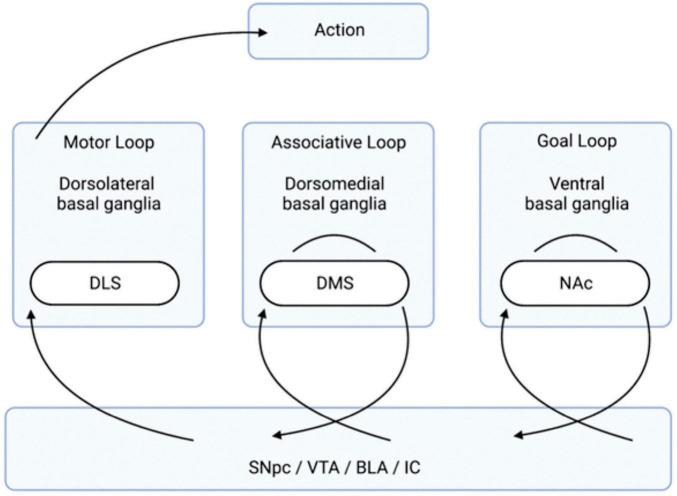
Organization of the Basal Ganglia Loops Involved in Goal-Directed Behavior. The transition of information, via the neurotransmission of relevant anatomical regions, flows from the right to the left and leads to action. Stimuli, creating an internal state of reward, come from the four listed brain regions below (the SNpc, VTA, and BLA/IC). The “goal loop” shows the interaction between the ventral basal ganglia (which contains the NAc) and the prefrontal cortex. The “associative loop” shows the interaction between the dorsomedial basal ganglia (which contains the DMS) and the PPC/PFC. The “motor loop” shows the interaction between the dorsolateral basal ganglia (which contains the DLS) and the motor cortex. BLA, basolateral amygdala; DLS, dorsolateral striatum; DMS, dorsomedial striatum; IC, insular cortex; NAc, nucleus accumbens; PFC, prefrontal cortex; PPC, posterior parietal cortex; SNpc, substantial nigra pars compacta; VTA, ventral tegmental area.

Research since the 1980s has expanded on this understanding to include a variety of loops that the basal ganglia participate in, many of which are not related to motor function (Lanciego et al., 2012). The basal ganglia loops relevant to this review are the ones that have an influence on goal-directed behavior and transitions to habitual control. Namely, there are three basal ganglia-cortical networks: 1) goal loop, 2) associative loop, and 3) motor loop (Figure 1; Mannella et al., 2016). First, the “goal loop” which is composed of loops involving the ventral basal ganglia (containing the NAc) and the prefrontal cortex (PFC) (Yin and Knowlton, 2006; Baldassarre et al., 2013; Mannella et al., 2016). The second, the “associative loop” which is composed of dorsomedial basal ganglia (containing the DMS) and posterior parietal cortex (PPC) (Yin and Knowlton, 2006; Baldassarre et al., 2013; Fiore et al., 2014; Mannella et al., 2016). The third, the “motor loop” which is composed of the dorsolateral basal ganglia (containing the DLS) and motor cortex (Yin and Knowlton, 2006; Baldassarre et al., 2013; Fiore et al., 2014; Mannella et al., 2016). This basic basal ganglia architecture creates the framework for assessing potential goal values, selecting them, and taking actions to pursue said goals (Belin et al., 2009; Passingham and Wise, 2012; Mannella et al., 2013, 2016; Mannella and Baldassarre, 2015). The question of where stimuli are initially associated with valence is answered in the amygdala complex (AC) (Mannella et al., 2016).

The AC, which includes more than 10 different nuclei, is the main anatomically relevant structure in the representation of stimuli and reward values, and the association of these values with internal body states (Sah et al., 2003; Mannella et al., 2016). Specifically, the basolateral amygdala (BLA) is notable in its contribution to reward processing as evidenced by experimentation assessing instrumental devaluation effects (IDEs) (West et al., 2012; Mannella et al., 2016). IDEs are measured whenever a mouse, or other experimental participant, presses two different levers for two distinct rewards over three phases. In the “instrumental phase” the mouse simply presses the levers and receives the rewards. In the “satiation phase” the mouse is given an excess of one of the rewards. In the crucial third phase, the “devaluation test,” the mouse is again given the two levers, but they are in extinction where no reward is given. IDE is observed when the mouse presses the lever associated with the scarce reward more than the satiated reward during this test (Mannella et al., 2016). IDE is thus a fair assessment for analyzing reward processing and goal-directed behavior.

When the BLA is transiently inactivated during the satiation stage, evidence shows that IDE disappears, but if it is inactivated after the satiation phase IDE remains (West et al., 2012). The gustatory region of the insular cortex (IC) is tightly connected to the BLA, and similarly is suggested to play a role in reward value registration and storage (Parkes and Balleine, 2013). It is currently posited that the BLA is needed for updating incentive values during satiation, and that the IC is needed for storage and retrieval of values during the crucial third phase devaluation test (Parkes and Balleine, 2013; Mannella et al., 2016). Connections between these two regions are not well understood, and thus the regions are referenced as a unit in their role of sending experienced, and then predicted, outcome values to the basal ganglia for goal selection, action, and motivation (Mannella et al., 2016).

The major recipient of these signals is the NAc (Mannella et al., 2016). Specifically, the NAc serves as a bridge for information from the BLA/IC to the ventromedial PFC (vmPFC) (Zahm, 2000). Whenever the NAc is lesioned IDE is prevented, as seen when the BLA/IC are lesioned (Corbit et al., 2001). This situates the basic entrance of reward value and stimuli information into the reward processing network as a simple two-step process from the BLA/IC to the NAc. In addition to that, a parallel network involving dopaminergic transmission from the VTA to the NAc carries information regarding the value of rewards (Mannella et al., 2016). Once that information is in the NAc, it is now within the aforementioned “goal loop.” From here, this portion of the basal ganglia sends signals to the ventral basal ganglia, then to the PFC, where signals are sent to the “associative loop” and reciprocally back to the NAc (Mannella et al., 2016). Importantly, NAc-PFC connections are where goals are differentially activated and action is thus biased (Mannella et al., 2013, 2016). This differential activation is mediated by the representation of internal states and reward values mediated by the BLA/IC-NAc and VTA-NAc connections (Cardinal et al., 2002; Passingham and Wise, 2012; Mannella et al., 2016).

For the selection, comparison, and motivation of goals to take place, the information from the “goal loop” must get to the associated loop, and to the “motor loop,” allowing IDE to take place. As previously mentioned, NAc lesions before or after learning prevent IDE from taking place. Intriguingly, when the prelimbic cortex, which exists within the mPFC and is referred to as prelimbic cortex (PL), is lesioned IDE is only prevented if those lesions take place before learning (Corbit and Balleine, 2003; Ostlund and Balleine, 2008; Tran-Tu-Yen et al., 2009). This suggests that information from the “goal loop” is transferred to motor and associative loops via some other pathway that links the NAc with those respective brain regions. In other words, reward values that need to be stored and processed in order to inform new behavior can be fed into the decision-making system without the participation of the PL, if the PL is present for the initial experience. After that, the information can travel through other neural pathways and behavior can be influenced without participation of the PL. The SNS spirals are excellent candidates for this function (Mannella et al., 2016).

**FIGURE 2:**
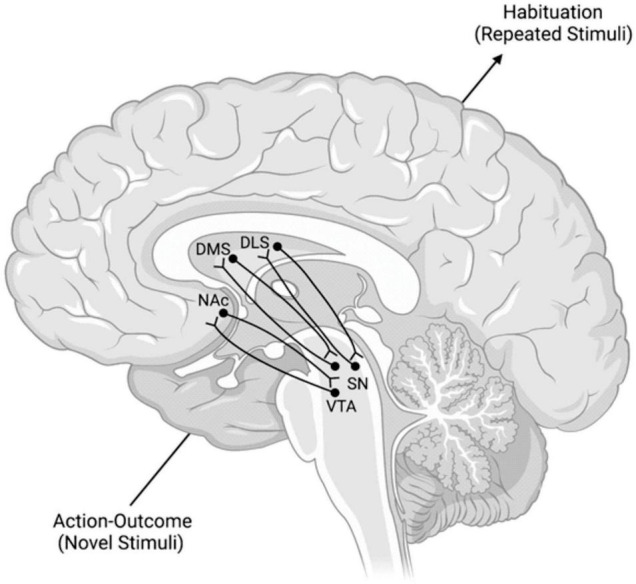
Model of the SNS Spirals and the Transmission of Information Within Them. The lower left dopaminergic projections from the VTA synapse in the NAc. NAc neurons then project back to a more dorsal region of the VTA. This pattern repeats in a ventral → dorsal and medial → lateral gradient from lower left to upper right. These spirals are posited to mediate the transition from action-outcome processing of novel stimuli to the habituation processing that occurs with repeated stimuli. DLS, dorsolateral striatum; DMS, dorsomedial striatum; NAc, nucleus accumbens; SN, substantia nigra; VTA, ventral tegmental area.

These spirals involve the VMS, the central striatum (CS), the DLS, and the dopaminergic neuronal projections of the VTA and substantia nigra pars compacta (SNpc) (Fudge and Haber, 2000; Haber et al., 2000; Haber, 2003). Each spiral is made of three SN components: a ventral component that receives a specific striatonigral projection but does not contain a reciprocal nigrostriatal projection, a central component that contains nigrostriatal and striatonigral reciprocal projections, and a dorsal component of just a nigrostriatal projection (Fudge and Haber, 2000; Haber et al., 2000; Haber, 2003). Each of these three components exists at each striatal position, within the VMS, CS, and DLS (Fudge and Haber, 2000; Haber et al., 2000; Haber, 2003). Thus, one can imagine a series of nine individual locations. These locations are separated into three groups, with each group containing one recipient area, one receiving and sending area, and one sending area. Information travels from one area to the next through these individual regions, ventral to dorsal and medial to lateral, and constitutes the SNS spiral network (Figure 2; Fudge and Haber, 2000; Haber et al., 2000; Haber, 2003).

These SNS spirals are proposed to operate by taking information from the NAc (a major component of the VMS) to the DMS (a major component of the CS) and then to the DLS of the associative and motor loops, respectively (Mannella et al., 2016). These connections interact with the VTA and SNpc, respectively (Mannella et al., 2016). This interface between midbrain areas, like the VTA and SNpc, and striatal areas, like the NAc, DMS, and DLS, encompasses the dopaminergic transmission of these spiral networks (Haber et al., 2000). The overall function of these spirals is suggested to be transferring goal values from the NAc to the DMS and the DLS, importantly without the involvement of the PL. This affords the basal ganglia an intrasystem mechanism for comparing and selecting goals without the involvement of the PL after learning has already taken place. In essence, these spirals are suggested to help the shift from action-outcome based processing of the DMS to stimulus-response based processing of the DLS without prelimbic cortical influence.

## Neurotransmitter Systems Related to SNS Spirals

### Dopamine

There are five dopamine receptors in the brain, D1–D5, which are categorized into D1-like and D2-like groups (Beaulieu et al., 2014; Misganaw, 2021). The D1-like receptors include D1 and D5, while the D2-like include D2–4 (Beaulieu et al., 2014). Dopamine projections come mainly from the VTA and SN (Beaulieu et al., 2014; Baik, 2020). The mesolimbic dopamine pathway is made up of dopaminergic projections from the VTA to the NAc (Cheron and Kerchove d’Exaerde, 2021). This midbrain to striatal pathway is a centerpiece to drugs of abuse, with virtually every abused drug stimulating dopaminergic transmission from the VTA to the NAc (Chiara and Imperato, 1988; Pontieri et al., 1996; Tanda et al., 1997). Not only that, but it is a component of the SNS spirals (Haber et al., 2000). The NAc shell projects to the VTA and the VTA projects back to the NAc shell and NAc core (NAcc) (Haber et al., 2000; Belin and Everitt, 2008).

These striatal projections to the midbrain are predominantly GABAergic, likely targeting GABA-B receptors (Edwards et al., 2017). In contrast, the VTA interneurons primarily target GABA-A receptors (Edwards et al., 2017). Interestingly, baclofen is a GABA-B receptor agonist that has been shown to reduce cue-associated cocaine craving and use in humans (Vocci and Elkashef, 2005; Young et al., 2014). It is suggested that this mechanism of SNS spiral pharmacologic intervention via GABA-B agonism from the striatum to the midbrain is what accounts for the observed therapeutic benefits (Edwards et al., 2017).

95% of the striatum’s projecting neurons are medium spiny neurons (MSNs) which are either D1 or D2 based on their genetic expression (Gerfen and Surmeier, 2011). More than just a large population, evidence shows that goal-direct learning differentially increases the activity of direct pathway D1 MSNs in IDE experiments (Shan et al., 2014). These changes were observed specifically in the DMS (Shan et al., 2014). The indirect pathway D2 MSNs did not experience that same increase in activity with the goal-directed behavior (Shan et al., 2014). Further inquiry into changes in synaptic plasticity, as measured by AMPA/NMDA ratio, showed sharp contrast between the increased ratio found in D1 MSNs in the DMS and the reduced ratio in D1 MSNs in the DLS (Shan et al., 2014). While these findings emphasize dopamine’s role in the striatum, particularly when it comes to goal-directed learning, recent research has delineated the influence of dopamine transmission on reward prediction error (RPE) (Lerner et al., 2021).

RPE is the error, or difference, in experienced vs. expected reward that an animal receives after a certain behavior and it was first hypothesized to be facilitated by dopaminergic transmission in 1997 (Schultz et al., 1997). Recent experiments utilizing optogenetic inhibition of VTA dopamine neurons revealed that these neurons encode the RPE alone, not the actual prediction itself (Maes et al., 2020). Interestingly, investigation into the mechanism with which dopaminergic neurons in the midbrain store RPE suggests that a distributional approach is taken (Dabney et al., 2020). In other words, rather than having each neuron register the same mean RPE, individual neurons will have variously pessimistic and optimistic RPEs for the brain to capture the full probability distribution to assist the learning process (Dabney et al., 2020). Other research suggests that the relative and total values, the predictions themselves, are maintained in the mPFC projections to the DMS, which coincides with other findings regarding the involvement of the DMS in goal-directed behavior (Bari et al., 2019).

Further underscoring the influence of dopamine in SNS spirals, disconnecting the NAcc from the DLS via the unilateral and bilateral blockade of dopamine receptors decreased cocaine-seeking behavior in rats (Vanderschuren et al., 2005; Belin and Everitt, 2008). One way the brain maintains motivation for rewards is the utilization of dopamine “ramps” (Lerner et al., 2021). These dopamine ramps consist of increased dopamine activity as an animal approaches their reward, serving to motivate the behavior even further (Lerner et al., 2021). The phenomenon has been observed in the VTA, ventral striatum, and DMS with the majority occurring during instrumental rather than Pavlovian tasks (Kim et al., 2020; Lerner et al., 2021). Not surprisingly, evidence shows relatively less ramping activity in the DLS, which has been shown to be more closely related to habitual rather than goal-directed behavior (Howe et al., 2013; Seiler et al., 2020).

**FIGURE 3:**
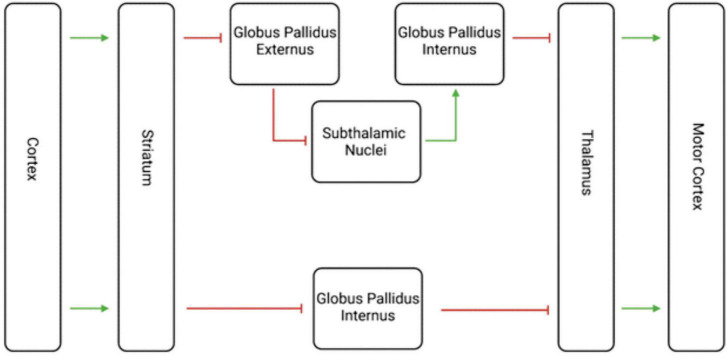
Visualization of the Indirect and Direct Motor Pathways. This diagram shows both the indirect and direct motor pathways, with the indirect motor pathways on top and the direct motor pathway below. The green arrows represent excitatory glutamate transmission, and the red lines represent inhibitory GABA transmission. The net effect of the indirect path is inhibition, while the net effect of the direct pathway is excitation. The striatum thus plays an important role in motor movement as well as decision making.

All these findings together place dopamine firmly in the center of the striatal and midbrain connections involved in SNS spirals which relate to goal-directed behavior. Dopamine is the neurotransmitter with which the midbrain modulates the striatum within this circuit (Volkow and Morales, 2015). However, many other neuron types exist within or affect the striatum, including glutamate, gamma-aminobutyric acid (GABA), acetylcholine (ACh), serotonin, cannabinoid, protons, and others (Pettibone et al., 1978; Volkow and Morales, 2015). These other neurotransmitter systems play roles in the many functions of the striatum, one of which is the mediation of motor movement (Packard and Knowlton, 2002). The two most notable circuits related to this function are the direct and indirect motor pathways (Figure 3; Purves and Williams, 2001).

### Glutamate and Gamma-Aminobutyric Acid

These two pathways are born out of medium spiny projection neurons from the striatum and are purported to have opposing effects on movement (Cui et al., 2013; Wang et al., 2015). Glutamate and GABA are the main excitatory and inhibitory neurotransmitters in the brain, respectively (Nahar et al., 2021; Niedzielska-Andres et al., 2021). The direct pathway’s striatal neurons receive excitatory glutamate transmission from the cortex, then send inhibitory GABAergic transmission to the globus pallidus internal segment (Knierim, 2020). That segment then sends inhibitory GABAergic transmission to the thalamus, which finally sends excitatory glutamatergic transmission back to the cerebral cortex (Knierim, 2020). The net effect of the direct pathway is disinhibition, or excitation, of movement via glutamate and GABA.

The indirect pathway similarly begins with excitatory glutamate transmission from the cerebral cortex via glutamate to the striatum, which then transmits inhibitory GABAergic transmission to the globus pallidus external segment (Knierim, 2020). Here, the external segment sends inhibitory GABAergic transmission to the subthalamic nuclei which themselves send excitatory glutamatergic transmission to the globus pallidus internal segment (Knierim, 2020). Finally, the internal segment similarly sends inhibitory GABAergic transmission to the thalamus, which transmits glutamatergic excitatory signals to the cerebral cortex (Knierim, 2020). The net effect of the indirect pathway is inhibition, via glutamate and GABA transmission.

These two pathways together facilitate motor learning, and this portion of their activity is most relevant to this review. In order to propagate a specific motor routine, the direct pathway must be increased while the indirect pathway has to be suppressed (Purves and Williams, 2001; Reiner et al., 2010). Driving to a new job would occur with concomitant direct pathway activation, and indirect pathway suppression. Where that drive to work becomes habitual, allowing the driver to zone out on the way home from a long day, is where the SNS spirals and dopaminergic transmission likely make an impact (Reiner et al., 2010).

A substantial collection of evidence supports the conclusion that dopamine signals interact with these motor systems to reward specific actions, like driving the correct route and successfully making it to work on time. Thus, this sculpts the striatal neurons during motor movement (Satoh et al., 2003; Graybiel, 2005; Reiner et al., 2010). In fact, dopamine receptor type one (D1) dependent long-term potentiation (LTP) has been demonstrated in direct pathway neurons, further supporting the idea that dopamine transmission interacts with glutamate and GABA transmission in the reward-based learning of behaviors (Kreitzer and Malenka, 2008; Shen et al., 2008; Reiner et al., 2010).

Outside of motor involvement, evidence suggests that GABA and other neurotransmitters have demonstrable influences within the striatonigral and nigrostriatal systems. Research involving the injection of kainic acid, a substance that is neurotoxic to striatal cholinergic and GABAergic nerves while leaving nigrostriatal dopaminergic nerve projections intact, reduces feeding and drinking in mice (Schwarcz and Coyle, 1977; Pettibone et al., 1978). This suggests that ACh and GABA neurons have an influence on behavior outside of dopaminergic signals (Schwarcz and Coyle, 1977; Pettibone et al., 1978). More recent evidence shows that these GABAergic MSNs modulate the midbrain neurons within the SNS circuit, influencing behavior (Yager et al., 2018; Yang et al., 2018). This provides the next phase of the SNS spiral after having been affected by the dopaminergic midbrain projections.

### Acetylcholine

The striatum is one of the most ACh dense regions of the brain (Izzo and Bolam, 1988). Striatal ACh is predominantly from intrastriatal cholinergic interneurons (ChIN), but there are two external sources in the pedunculopontine and laterodorsal tegmental nuclei (Dautan et al., 2014; Abudukeyoumu et al., 2018; Lindroos and Kotaleski, 2020). These ChINs synapse onto the MSNs of the indirect and direct pathways (Abudukeyoumu et al., 2018). Input from these neurons is neuromodulatory in nature, often working in tandem with the modulatory dopamine transmission (Lindroos and Kotaleski, 2020). Fast scan voltammetry showed that simultaneous activation of ChINs led to an increase in striatal dopamine release (Cachope and Cheer, 2014). Additionally, endogenous release of ACh has been observed to directly increase striatal dopamine release (Cachope et al., 2012). Functionally, this interneuron ACh transmission has been posited as a switch between two modes of dopamine transmission, its action as learning stimulus and as a motivational cue (Berke, 2018). In regard to motor activity, both ACh and dopamine are necessary for the induction of locomotion (Zucca et al., 2018).

The striatum itself was discovered to contain two complementary chemical compartments with unique genetic expression, striosomes and the surrounding matrix (Graybiel and Ragsdale, 1978; Herkenham and Pert, 1981; Graybiel and Hickey, 1982; Crittenden and Graybiel, 2011). The striosomes were shown to have reduced cholinesterase activity in adult humans, rhesus monkeys, and cats (Graybiel and Ragsdale, 1978; Crittenden and Graybiel, 2011). These acetylcholinesterase-poor striosomes were found to have over 40 different enriched genes, including being enkephalin-rich, while the matrix regions were found to have over 20 different uniquely enriched genes (Graybiel and Hickey, 1982; Crittenden and Graybiel, 2011). These genetic differences have clinical correlates in disease, where the striosome to matrix ratio of immediate early gene (IEG) induction increases in cocaine and psychomotor stimulant addiction in monkeys and rats, respectively (Hurd and Herkenham, 1993; Moratalla et al., 1996; Crittenden and Graybiel, 2011). Importantly, evidence suggests that striosomes contain the only striatal neurons that project to the SNpc, underpinning their involvement in SNS spirals (Gerfen, 1984; Jiménez-Castellanos and Graybiel, 1989; Tokuno et al., 2002; Crittenden and Graybiel, 2011; Fujiyama et al., 2011). Thus, ACh plays an important role in modulating striatal neurons as they participate in the greater SNS circuit.

### Opioids

Related to the acetylcholinesterase-poor quality of striosomes is their enkephalin-rich quality, and the role of opioids in the striatum in general. While glutamate and GABA are the principal molecules of the motor pathways, with dopamine involved in the learning component of motor activity, endogenous opioids are paired co-transmitters in the respective direct and indirect pathway (Steiner and Gerfen, 1998; Hearing, 2019; Koob, 2020). Specifically, D1 receptor expressing cells of the direct pathway express dynorphin while D2 expressing cells of the indirect pathway express enkephalin (Steiner and Gerfen, 1998). Interestingly, neurons that express both substance P and enkephalin have been found in the striatum, with a higher proportion in the striosome regions (Besson et al., 1990; Wang et al., 2007). Following suit with the impression that SNS spirals are significant, co-expression of substance P and enkephalin are found in the SNpc, but not in the substantial nigra pars reticulata (SNr), globus pallidus internus (GPi), or globes pallidus externes (GPe) (Wang et al., 2006). This insinuates that a unique set of D1 and D2 expressing neurons exist in the striatum, and that a unique set of opioid expressing neurons are evident in the SNpc projection component of the SNS spirals. Recent evidence examining opioid expressing neurons in the SNS circuit found that roughly 50% of GABA neurons in the SNr have μ-opioid receptors (MORs) which, when activated, lead to the disinhibition of SNpc DA neurons and the processing of reward with drugs like heroin (Galaj and Xi, 2021). However, because the opioid and dopamine receptors are subject to expression change based on the experience of the being, further investigation is needed to examine this possibility (Steiner and Gerfen, 1998; Crittenden and Graybiel, 2011; Hearing, 2019).

### Serotonin

Another neurotransmitter system which plays an important role in the striatum is serotonin (5-HT) (Bonsi et al., 2007; Robbins et al., 2019; Tong et al., 2020). Patients with Parkinson’s disease have shown proportional decreases in 5-HT transmission to the decrease in dopamine transmission, insinuating an involvement in the motor and affective activity (Sandyk and Fisher, 1988; Halliday et al., 1990). In addition, the use of selective serotonin reuptake inhibitors (SSRIs), namely for the treatment of depression, has been associated with movement symptoms like tremor, parkinsonism, and dystonia (Leo, 1996; Caley, 1997). Interestingly, serotonergic transmission is excitatory to the ChINs (Bianchi et al., 1989; Bonsi et al., 2007). This relationship between serotonin and ACh, paired with ACh density in striosomes that are involved in SNS spirals, sheds light on a potential relationship with yet another neurotransmitter system and the striatonigrostriatal pathways.

### Cannabinoids

Cannabinoid receptors play a significant role in the striatal system (Piomelli et al., 1999). In fact, there are twice as many cannabinoid receptors as D1 dopamine receptors and 12 times as many MORs in the striatum (Herkenham et al., 1991; Sim et al., 1996; Piomelli et al., 1999). Due to their interactions with the motor system, various cannabinoids like cannabidiol (CBD) have been shown to reduce seizure frequency in those with refractory epileptic encephalopathies (Morano et al., 2020). Outside of their influence on motor activity, cannabinoids have been shown to influence learning, habit formation, and addiction as well (Lovinger et al., 2010; Hoffman and Lupica, 2012; Davis et al., 2018). Functionally, endocannabinoids signal retrogradely via suppression of synaptic transmission through presynaptic G-protein coupled receptors (GPCR) like the cannabinoid-1 receptor (CB_1_-R) (Davis et al., 2018).

Within the striatal circuitry, these CB_1_-Rs modify dopamine signaling (Covey et al., 2017; Mateo et al., 2017; Davis et al., 2018). Interestingly, these receptors are found in a density gradient from least, ventromedial, to most, dorsolateral (Julian et al., 2003; Waes et al., 2012; Davis et al., 2018). This neuroanatomical layout matches data regarding CB_1_-Rs and their influence on behavior, specifically regarding transitions from goal-directed to habitual behavior (Yin et al., 2004; Hilário, 2007; Davis et al., 2018). Strikingly, this evidence is paired with further findings that show enrichments of CB_1_-Rs in striosome compartments within the striatum (Herkenham et al., 1991; Julian et al., 2003; Martín et al., 2007; Lovinger et al., 2010; Davis et al., 2018). Together, these data suggest that CB_1_-Rs play an important role in the SNS circuit via their influence on dopamine signaling (Guegan et al., 2015). These studies suggest that endogenous cannabinoids play a significant role within the striatum, within the transition to habitual behavior from goal-directed processing and are again found at higher densities in striosomal pockets like that of ACh.

### Proton

Recently, protons have been identified as a neurotransmitter in the brain (Du et al., 2014). After their release during neurotransmission, protons (e.g., pH drops) bind to their postsynaptic receptors named acid-sensing ion channels (ASICs) (Waldmann et al., 1997; Krishtal, 2015; Gobetto et al., 2021; Rook et al., 2021). These receptors are derived from the greater degenerin/epithelial sodium ion channel family, which mediate sodium influx across membranes in a voltage-insensitive manner largely responsible for neurological and psychological functions (Waldmann et al., 1997; Gründer and Pusch, 2015; Cheng et al., 2018; Vullo and Kellenberger, 2020). The ASIC1a, ASIC2a, and ASIC2b subtypes are found predominantly in the brain, with the ASIC1a subtype being the most densely populated in the striatum (Biagini et al., 2001; Alvarez de la Rosa et al., 2003; Wemmie et al., 2003; Jiang et al., 2009; Suman et al., 2010; Price et al., 2014; Krishtal, 2015).

Studies from our laboratory demonstrated that ASIC1a receptors were up-regulated in the mouse striatum but not in the mPFC in response to repeated cocaine exposure (Zhang et al., 2009). We also found similar results using amphetamine rather than cocaine in rats. The ASIC1a receptors were up-regulated after chronic amphetamine exposure in the rat striatum but not in the mPFC (Suman et al., 2010). The data also showed a reduction of ASIC2 expression in the mPFC (Suman et al., 2010). Further, we examined the behavior changes by repeated cocaine administration in ASIC1a and ASIC2 knockout (KO) mice. Behavioral sensitization to cocaine was seen in wild-type (WT) and ASIC1a KO mice, but not in ASIC2 KO mice (Jiang et al., 2013). Additionally, in ASIC1a KO mice, cocaine induced significantly fewer motor responses at varying doses compared to WT and ASIC2 KO mice (Jiang et al., 2013). Studies from other laboratories also revealed that ASIC1a in the amygdala and NAc contributes to cocaine addiction (Kreple et al., 2014; Gutman et al., 2020). Further examination of densities of these receptors in striosomes could provide more insight into their function. Together, these observations suggest that ASIC receptors help facilitate the process of reward and have an important relationship with the striatal architecture reviewed here.

The involvement of dopamine, glutamate, GABA, ACh, dynorphin, enkephalin, serotonin, cannabinoids, and protons in the striatal system alludes to the complex nature of the striatum and the SNS spirals themselves. An in-depth review of each one of these neurotransmitter systems and their impact on the reciprocal connections of the midbrain and striatum would be valuable additions to scientific literature. In the case of this review, a surface-level inquiry into each is sufficient to emphasize the point that SNS spirals are playing an elaborate role in the motor, goal-oriented, and habitual behavior mediated by the SNS network.

## Striato-Nigro-Striatal Spirals and Addiction

As mentioned earlier, drugs of abuse facilitate dopaminergic transmission from the VTA to the NAc (Chiara and Imperato, 1988). The NAc then participates in the SNS spirals where information is transferred from the goal loop, to the associative loop, and then to the motor loop. Specifically, neurological activity during goal-directed behavior pairs with activity in the DMS, while habitual behavior pairs with activity in the DLS (Belin et al., 2009; Redgrave et al., 2010). This process of transfer from goal to habit vaguely mirrors the experience of a first-time reward becoming an inherent desire, like trying your favorite ice cream for the first-time and eventually having that flavor come to mind every time you think of ice cream. But this isn’t specific to addiction, and instead is an essential component of human life.

Further underlying this tenant that the DLS facilitates habitual behavior, and the DMS facilitates goal-directed behavior, is the fact that goal-directed behavior is retained after lesions to the DLS (Yin, 2004; Yin et al., 2004; Yin and Knowlton, 2006; Rossi and Yin, 2012; Lipton et al., 2019). Expectedly, lesions to the DMS lead to the early formation of habitual behavior considering the DLS is preserved (Yin et al., 2005; Yin and Knowlton, 2006; Lipton et al., 2019). These foundational findings inspired more investigation, leading to the observation of unique patterns of activity in the DLS (Lipton et al., 2019). Researchers found that a mouse running a maze will have a high level of neural activity in the striatum the first time, but over multiple attempts activity in the brain, specifically in the DLS, begins to maximize at the beginning and end of runs (Jog, 1999; Barnes et al., 2005; Thorn et al., 2010; Smith and Graybiel, 2013; Graybiel and Grafton, 2015). Activity during the run decreases, sometimes reaching levels even lower than pre-run baselines (Lovinger et al., 2010). Experiments have also observed this phenomenon in lever-pressing tasks and in macaque monkeys during well-learned motor skills (Fujii, 2003; Jin and Costa, 2010; Martiros et al., 2018). This is termed task-bracketing.

The mechanism of how the SNS circuit relates to task-bracketing is an important open question. One group examined this observed striatal firing pattern and aimed to distinguish it from motor cortex firing patterns that may have explained the striatal activity (Martiros et al., 2018). Their findings showed that striatal projecting neurons and fast spiking interneurons (FSI) in the DLS have unique activity compared to the motor cortex, and are key to habitual behaviors (Martiros et al., 2018). These interneurons are the same GABAergic interneurons within the SNS circuit that mediate transitions of information within the striatum (Haber et al., 2000). Another study used chemogenetic inhibition of these FSI and found that disruption of their activity reduced the ability to express habitual behavior (Ohare et al., 2017). The authors clarified that these interneurons are important because of their specific influence on striatal output properties and their long-lasting changes in excitability following habituation (Ohare et al., 2017). Findings in Martiros et al. (2018) and Cunningham et al. (2021) distinguished this task-bracketing from the actual reward processing of correct lever presses and suggested instead that this firing pattern in the DLS is a neuronal representation of successful past behaviors. This theoretical framework aligns with previous statements regarding the SNS circuit’s role in shifting activity from goal-directed behavior to habitual behavior with past actions as reference points.

The question then arises, if the task-bracketing activity pattern in the DLS represents past actions with their paired outcomes, how is the SNS circuit posited to facilitate the transfer from NAc to DLS activity. One study examined this directly using reversible neurotransmission blockade (RNB) of D1 or D2 MSNs in the NAc, DMS, and DLS via tetanus neurotoxin in mice (Macpherson and Hikida, 2018). They found that NAc D1 RNB mice had reduced Pavlovian approaches to conditioned stimuli, while NAc D2 RNB, DMS, and DLS RNB mice did not (Macpherson and Hikida, 2018). Specifically, the authors stated, “blockade of neurotransmission in NAc D1 MSNs appears to reduce the transference of incentive salience from the liquid reward to the conditioned stimulus.” These data support the position that early in the SNS circuit, midbrain projections to DA MSNs in the NAc must be activated for incentive salience to be transferred to conditioned stimuli (Macpherson and Hikida, 2018). One could then hypothesize that SNS spirals are necessary pieces of the neural framework which allows for conditioning, and eventually habituation (Flagel et al., 2010; Peak et al., 2018; Lipton et al., 2019). With NAc activation, NAc reciprocal projections to the VTA and SNpc modulate neurons which project to the more dorsal striatum (Haber et al., 2000; Mannella et al., 2016). Thus, the continuous activation of VTA and SNpc DA neurons, via the SNS circuit, help to facilitate habituation which at least in part is represented by DLS task-bracketing activity (Mannella et al., 2016; Martiros et al., 2018; Cunningham et al., 2021).

A notable contrast to the task-bracketing observed in the DLS is the almost exactly opposite pattern evident in the DMS (Julian et al., 2003). Here, neural activity is enhanced during the middle of an action in comparison to the beginning and end, especially during novel activities (Yin et al., 2009; Thorn et al., 2010; Gremel and Costa, 2013; Martiros et al., 2018; Lipton et al., 2019). Overtime, as the task-bracketing of the DLS occurs, this DMS phenomenon fades away (Yin et al., 2009; Gremel and Costa, 2013; Martiros et al., 2018; Lipton et al., 2019). Viewing these neural activation schemes as mutually exclusive isn’t entirely accurate. More evidence suggests that these two firing patterns either cooperate or compete with one another for dominance over behavior (Daw et al., 2005; Yin and Knowlton, 2006; Bradfield and Balleine, 2013; Gremel and Costa, 2013; Kupferschmidt et al., 2017; Robbins and Costa, 2017; Lipton et al., 2019). When the DLS is intentionally inactivated after habitual behavior has been established, goal-directed behavior is reestablished (Yin and Knowlton, 2006). This can be utilized in some sense to improve learning in early training via optogenetic silencing or DLS lesions (Bradfield and Balleine, 2013; Bergstrom et al., 2018). These experimental observations further underscore the importance of this transition from goal-directed to habitual behavior.

Studies specific to addiction have substantiated these conclusions (Corbit, 2018; Lipton et al., 2019). This path of goal directed DMS activity to habitual DLS activity continues to show up in experimentation. In the beginning of consumption, drug-seeking is found to be goal-directed and mediated by the DMS amongst a larger network (Corbit et al., 2012; Murray et al., 2014; Lipton et al., 2019). Intriguingly, inactivation of the DLS in cocaine addicted rats discontinues punishment-resistant seeking of drug-predicting cues (Jonkman et al., 2012; Lipton et al., 2019). Additionally, rats that are trained to press levers for a cocaine reward will minimize their lever pressing following dopamine antagonists in the DMS during the beginning of the learning process, or in the DLS during the over-training component of the experiment (Vanderschuren et al., 2005; Murray et al., 2012; Lipton et al., 2019). This same experiment modality was replicated with lidocaine induced DLS inactivation (Zapata et al., 2010; Lipton et al., 2019). Alcohol was also found to disinhibit the spiny projection neurons in the DLS, which could serve as a conduit for the transfer from goal-directed to habitual use (Wilcox et al., 2013; Patton et al., 2015; Lipton et al., 2019). Additionally, DLS activity has been found to be imperative for habitual heroin seeking in rats (Hodebourg et al., 2019; Lipton et al., 2019). In total, there is sufficient evidence to defend the claim that addiction is built on neural circuitry that involves a transfer of activity from the DMS to the DLS.

If this DLS-mediated habitual behavior was extinguishable with ease, addiction might not be the problem it is today. However, even in devaluation experiments which can make the reward essentially unpalatable, there are no observed changes to the task-bracketing activity in the DLS (Smith and Graybiel, 2013; Graybiel and Grafton, 2015). In order to remove this task-bracketing activity in the DLS, the reward would have to be entirely removed, and even then, when it is reintroduced or cues are presented, the same activity resumes (Graybiel and Grafton, 2015). Projections to the striatum are not solely responsible for this formidable DLS activity, the intrastriatal SNS components play an active role as well. These connections, components of the SNS spirals like the previously mentioned ChINs and related striosomes, are integral (Smith and Graybiel, 2013; Atallah et al., 2014; Graybiel and Grafton, 2015). All together, these findings suggest that some neural circuits, likely including SNS spirals, facilitate this transfer from goal-directed to habitual processing and sustain it over time.

Though these striatal changes in activity that take place during habituation are significant, concomitant changes in SN and VTA neurons in addiction must also be observed. The substantia nigra pars reticulata (SNpr) has GABAergic neurons which form reciprocal connections with SNpc DA neurons (Galaj and Xi, 2021). A recent study found that roughly 50% of GABA neurons in this region have MORs which, when activated, lead to the disinhibition of SNpc DA neurons and the processing of reward with drugs like heroin (Galaj and Xi, 2021). Optogenetic stimulation of these SNr neurons, or of the SNpc DA neurons directly, leads to rewarding effects (Galaj et al., 2020). Interestingly, high frequency deep brain stimulation (DBS) of the SNpr of rats blocked drug-primed reinstatement and manifested the extinction of methamphetamine-induced conditioned place preference (CPP) (Zhang et al., 2021). DBS, however, did not reduce the rewarding effects of methamphetamine administration (Zhang et al., 2021). The authors suggested that the DBS promoted extinction and prevented drug-primed reinstatement via induction of LTP within the SNpr that decreased activity in the dorsal striatum (Zhang et al., 2021). Zhang et al. (2021) go on to say “thus, modulating the activity of SNpr may regulate addiction by affecting striatum activity.” These data correspond with the previous point that the DLS is a center point of habituation, and earlier portions of the SNS circuit are involved in the preceding goal-directed reward processing (Lipton et al., 2019).

Another recent study showed that optogenetic stimulation of SNpc DA neurons produced real-time place preference and optical intracranial self-administration (iOCSS) in TH-cre and DAT-cre mice at similar levels as VTA DA neurons (Galaj et al., 2020; Galaj and Xi, 2021). In fact, inhibition of SNpc DA neurons or VTA DA neurons can induce aversion (Ilango et al., 2014; Galaj et al., 2020). Interestingly, even the removal of aversive stimuli leads to very different SNpc activity depending on whether these changes occur during learning or habituation (Diao et al., 2021). VTA DA neurons have been found to increase their firing rate following chronic morphine treatment (Simmons et al., 2019). Cocaine administration increases the AMPA/NMDA ratio in VTA DA neurons that project to either the NAc core or shell but not in those VTA DA neurons projecting to the PFC (Lammel et al., 2011). These data show the importance of midbrain DA neurons projecting to the striatum, and the changes that occur during the pathology of addiction. Integration of these two midbrain regions in addiction is posited to take place via dendrodentritic DA connections, activity in the rostromedial tegmental nucleus, or via activity in the striatum (Galaj and Xi, 2021). It is this review’s position that the striatum is the most influential of the three.

To underscore that position, further evidence suggests that changes in striatal neurons coincide with these relevant midbrain changes. Decreasing activity of direct pathway GABAergic MSNs reduced drug-seeking in a high-risk mouse phenotype during cue-induced reinstatement, without altering behavior in a low-risk phenotype (Yager et al., 2018). Optogenetic stimulation of terminals in the VTA which came from the lateral shell of the NAc successfully induced a potent reward response, which the authors attribute to disinhibition via these striatal neurons (Yang et al., 2018). With even one use of morphine, CB_1_-R KO mice had increased total dendritic spine density in the NAc shell and core suggesting both a regulatory role for CB_1_-R and distinguished striatal MSN changes with substance abuse (Guegan et al., 2015). More than just one exposure, repeated administration of morphine to establish CPP is prevented by the downregulation or antagonism of D1 dopamine receptor containing MSNs in the NAc and is perpetuated with agonism (Hearing et al., 2016; Kobrin et al., 2017; Thompson et al., 2021). There are even notable differences in how NAc MSNs are modified by cocaine and opioid use and their respective withdrawals (Graziane et al., 2016). Importantly, NAc MSNs are modified in both cases. Again, these data show that these neurons, which project to the midbrain, are changed in the pathology of addiction. This further situates the SNS circuit as a major pathway in the pathology of addiction.

## Discussion

This review sought to examine the neurobiology of SNS spirals and their relation to addiction. The anatomy and function of the basal ganglia which itself contains these spirals, other neurotransmitter systems’ interaction with these spirals, and specific experimental findings related to addiction were explored. Together, these pieces of evidence support the claim that SNS spirals are central to the transition from goal-directed to habitual behavior. A clinically relevant example is the transition from substance use to addiction.

In a very basic analogy, it was found that the dopaminergic transmission of reward can be likened to the money made by a company. The chief executive officer (CEO), or the PFC, is happy to make as much money as possible considering that is their responsibility to the company. Certain behaviors, like being socially accepted or rejected, can reduce or increase the amount of dopamine transmitted and money made. Through the factories of deeper brain components like the VTA, SNpc, and BLA/IC, these reward signals are profitably produced. Middle managers in the striatum receive information regarding the productivity of the various factories within the company, and associate values with specific behaviors. The CEO then receives a summary revenue report via projections from the striatum to the PFC.

**FIGURE 4:**
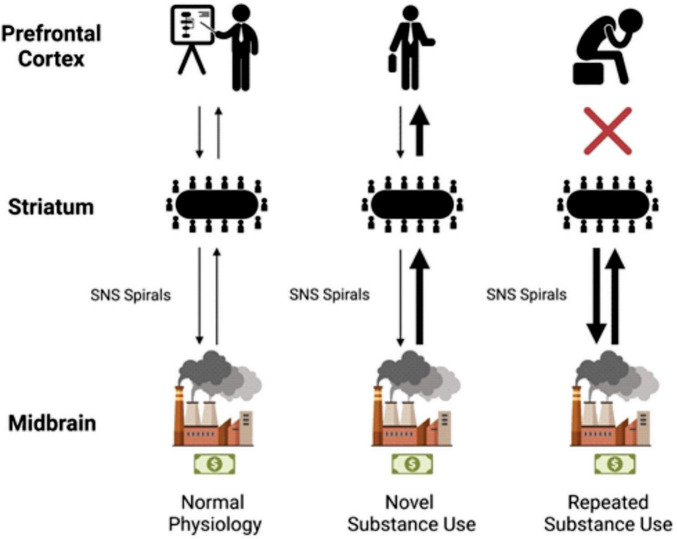
The left of the model shows anatomic labels. The “CEO” of the prefrontal cortex (PFC), the “middle managers” of the striatum, and the “factories” of the midbrain. In normal physiology, dopaminergic projections from the midbrain make their way to the striatum (represented by a thin black arrow). Further striatal activity facilitates this information getting to the PFC. Activity then flows back, and a general processing of rewards takes place. With novel substance use, a much larger dopaminergic transmission (represented by a thicker black arrow in the center column) flows to the striatum; the company makes more money than usual. The middle managers (in the striatum) are informed of the increase in profit from their factories. The PFC again is influenced by this transmission, the CEO learns of this new profitable venture, and a person experiences it. With repeated substance use, habituation takes place, the SNS spirals shift from ventral to dorsal and from medial to lateral utilizing the NAc, DMS, and then the DLS. The middle managers believe that this profitable venture is what’s best for the company regardless of the CEO’s protestations and decide to manage the factories on their own. CEO, chief executive officer; DLS, dorsolateral striatum; DMS, dorsomedial striatum; NAc, nucleus accumbens; PFC, prefrontal cortex; The first businessman icon was downloaded from Flaticon, the second businessman icon was created by Mundo from Noun Project.

Overtime, well-intentioned hard working middle managers decide that the CEO need not be bothered with information regarding well known profitable tasks. So, they take matters into their own hands and reduce the workload for their boss by utilizing SNS spirals. They transfer activity from the NAc and DMS centric domain to the DLS centric domain using the SNS spirals. Unknown to the middle managers and factories of the deeper brain networks, those previously fruitful actions land the CEO in hot water in the world of human affairs. With a new desire to avoid punishment, the CEO calls the middle managers and explains a discontinuation of the old money-making strategies. However, due to the determined work ethic of his trusted employees, his orders are not followed. After all, the middle managers ask themselves, how could we possibly stop our most profitable revenue stream after some seemingly mild resistance? Thus, the DLS task-bracketing remains formidable, even following executively mandated abstinence, and is at the ready following the proper cues. This can persist to such a degree that the CEO is eventually left out of company decisions altogether, and a coup d’état of sorts via SNS spirals leads to the deeper brain networks running the company. The harrowing struggles of addiction may surface, but a determined and mislead vision of metaphoric wealth will drive the reorganized institution into the ground one dopamine burst at a time (Figure 4).

While this analogy is incomplete and inaccurate at certain levels of detail, the general principle is sound. The PFC and the person who suffers from the disease of addiction lose much of their ability to choose to engage in the increasingly harmful behavior. It is the duty of science, and of humanity, to reduce those suffering by making use of the evidence we find. Therefore, therapeutic efforts targeted at these spirals and their facilitation of habituation should be sought. In fact, some researchers have already made use of contemporary technology like DBS to depotentiate excitatory synaptic inputs on dopamine D1 receptors, which mimics the practice of optogenetic metabotropic glutamate receptor-dependent normalization of synaptic transmission (Creed et al., 2015; Lüscher et al., 2015). While this won’t be sufficient to treat the pandemic facing the US and other parts of the world, a resilient and compassionate effort can be founded on this information.

## Author Contributions

All authors listed have made a substantial, direct, and intellectual contribution to the work, and approved it for publication.

## Conflict of Interest

The authors declare that the research was conducted in the absence of any commercial or financial relationships that could be construed as a potential conflict of interest.

## Publisher’s Note

All claims expressed in this article are solely those of the authors and do not necessarily represent those of their affiliated organizations, or those of the publisher, the editors and the reviewers. Any product that may be evaluated in this article, or claim that may be made by its manufacturer, is not guaranteed or endorsed by the publisher.
